# Crystal structure of copper perchlorophthalo­cyanine analysed by 3D electron diffraction

**DOI:** 10.1107/S2052520621006806

**Published:** 2021-07-29

**Authors:** Tatiana E. Gorelik, Stefan Habermehl, Aleksandr A. Shubin, Tim Gruene, Kaname Yoshida, Peter Oleynikov, Ute Kaiser, Martin U. Schmidt

**Affiliations:** a Electron Microscopy of Materials Science (EMMS), Ulm University, Albert Einstein Allee, 11, 89077 Ulm, Germany; b Institute of Inorganic and Analytical Chemistry, Johann Wolfgang Goethe University, Max-von-Laue-Str. 7, 60438 Frankfurt am Main, Germany; c Institute of Solid State Chemistry and Mechanochemistry SB RAS, Kutateladze, 18, Novosibirsk, 630128, Russian Federation; d Novosibirsk State University, 1, Pirogova str., Novosibirsk, 630090, Russian Federation; e Department of Inorganic Chemistry, University of Vienna, Waehringer Str. 42, Vienna, 1090, Austria; f Japan Fine Ceramics Center, 2-4-1 Mutsuno, Atsuta-ku, Nagoya, 456-8587, Japan; g Department of Physics, Science and Technology, ShanghaiTech University, Shanghai, People’s Republic of China

**Keywords:** electron crystallography, 3D electron diffraction, continuous rotation, copper phthalo­cyanine, Pigment Green 7, Rietveld refinement, DFT+MBD calculations

## Abstract

Copper perchlorophthalo­cyanine (CuPcCl_16_, CuC_32_N_8_Cl_16_, Pigment Green 7) is one of the commercially most important green pigments. The compound is a nanocrystalline fully insoluble powder. Its crystal structure was first addressed by electron diffraction in 1972 [Uyeda *et al.* (1972). *J. Appl. Phys.* **43**, 5181–5189]. Despite the commercial importance of the compound, the crystal structure remained undetermined until now. Using a special vacuum sublimation technique, micron-sized crystals could be obtained. Three-dimensional electron diffraction (3D ED) data were collected in two ways: (i) in static geometry using a combined stage-tilt/beam-tilt collection scheme and (ii) in continuous rotation mode. Both types of data allowed the crystal structure to be solved by direct methods. The structure was refined kinematically with anisotropic displacement parameters for all atoms. Due to the pronounced crystal mosaicity, a dynamic refinement was not feasible. The unit-cell parameters were verified by Rietveld refinement from powder X-ray diffraction data. The crystal structure was validated by many-body dispersion density functional theory (DFT) calculations. CuPcCl_16_ crystallizes in the space group *C*2/*m* (*Z* = 2), with the molecules arranged in layers. The structure agrees with that proposed in 1972.

## Abstract 2

The structure of copper perchlorophthalo­cyanine (CuC_32_N_8_Cl_16_, Pigment Green 7) was solved from three-dimensional electron diffraction data (3D ED) and placed into the series of known copper phthalo­cyanine crystal structures.

## 1. Introduction   

Copper phthalo­cyanine (CuPc, Scheme 1) is the most important blue pigment used today (Hunger & Schmidt, 2018 ▸). [Chem scheme1] 
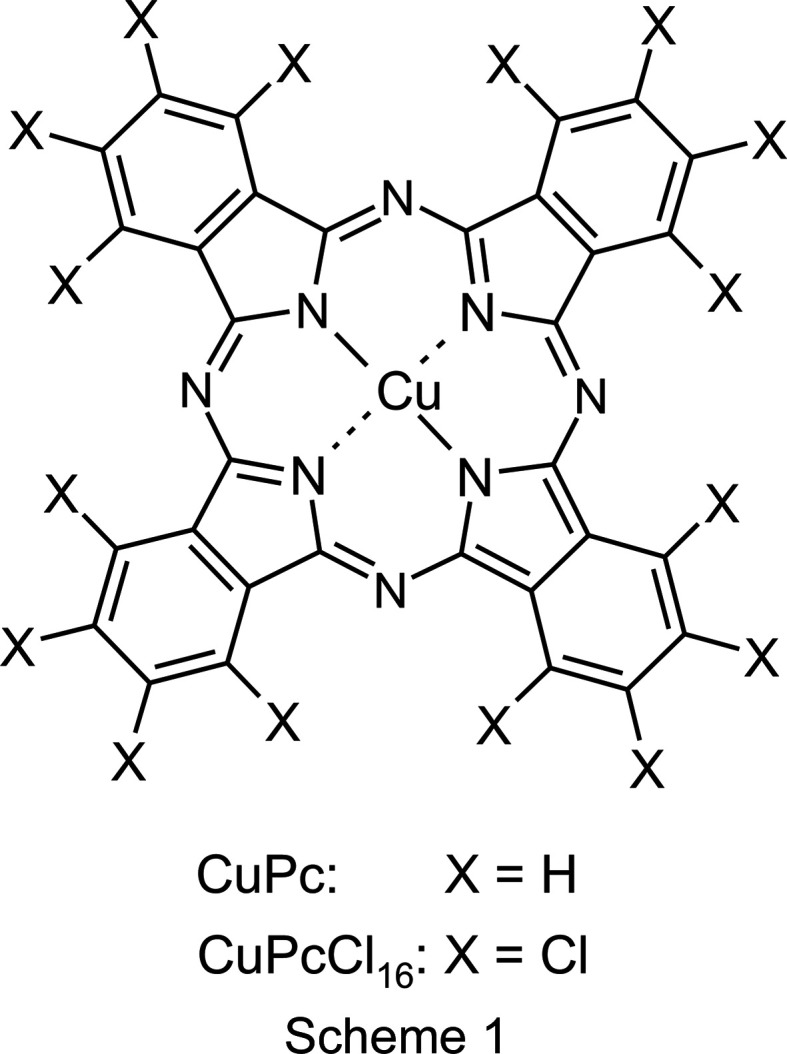


**Figure 1:**
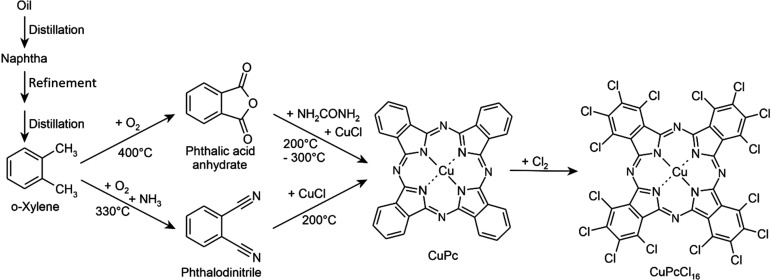
The synthesis of copper perchlorophthalo­cyanine.

Chlorination of CuPc shifts the colour towards green shades. The fully chlorinated CuPcCl_16_ compound shows a bright bluish green shade. CuPc and CuPcCl_16_ are registered in the Colour Index as Pigment Blue 15 and Pigment Green 7, respectively (Abel, 1998 ▸). Both pigments are widely used for the colouration of lacquers and coatings, paints, polymers of all kinds, printing inks, artists’ colours, office articles, wax, rubber, *etc*. The pigments exhibit an excellent photostability and, consequently, a very high fastness to light and weathering. Their heat stability is outstanding, to more than 500 °C, so that they can even be used for the mass colouration of low-melting glass (Schmidt & Kliemt, 2013 ▸). Their industrial synthesis is quite easy (see Fig. 1 ▸). Correspondingly, their price is quite low, only 5–10 Euro per kilogram.

CuPc was discovered by chance almost 100 years ago. In 1927, the Swiss chemists Henri de Diesbach and Edmond von der Weid attempted to synthesize phthalo­di­nitrile by reacting 1,2-di­bromo­benzene with CuCN in pyridine at 200 °C and obtained a blue copper com­plex of unknown structure. A year later, FePc was discovered by chance, too. In the reaction of phthalic acid anhydride with ammonia, chemists from Scottish Dyes Ltd observed a blue impurity on the enamel reactor vessel. Apparently, the enamel was defective, so that the reaction mixture could react with the vessel, and FePc was formed. They wrote a patent on the new compound mentioning that ‘the products do not appear to be metal salts of phthalimide’ (Dandridge *et al.*, 1928 ▸). A few years later, Linstead determined the correct molecular composition of the phthalo­cyanine com­plexes (Dent *et al.*, 1934 ▸). The commercial production of CuPc began in 1935 and that of CuPcCl_16_ in 1938 (Hunger & Schmidt, 2018 ▸).

CuPc is known to exist in at least ten poly­morphic forms (Erk & Hengelsberg, 2003 ▸). The industrially important phases are the α, β and ɛ phases. The hue of CuPc depends on the packing of the molecules in the solid state. The α-phase is blue with a slight reddish shade and the ɛ-phase is even more reddish. The β-phase is a slightly greenish blue, which matches the standard ‘cyan’ hue used for four-colour printing. β-CuPc is used worldwide for the printing of almost all newspapers, books and journals. *Acta Crystallographica*, when available in print form, was printed with β-CuPc, as we confirmed by mass spectrometry using the laser–desorption–ionization technique^1^. β-CuPc is also used in toners for laser printers and copying machines.

Metal-free phthalo­cyanine exists in seven poly­morphic forms (Bernstein, 2002 ▸).

For CuPcCl_16_, only one poly­morphic form is known.

Crystal structures are known for four phases of CuPc, *i.e.* α, β, γ and ɛ (Robertson, 1935 ▸, 1936 ▸; Brown, 1968 ▸; Moxon *et al.*, 1981 ▸; Ruiz-Ramirez *et al.*, 1987 ▸; Erk, 2002 ▸; Erk & Hengelsberg, 2003 ▸; Hoshino *et al.*, 2003 ▸; Erk *et al.*, 2004 ▸; Xia *et al.*, 2008 ▸; Wang *et al.*, 2008 ▸; Jiang *et al.*, 2018 ▸). The structure of the β-phase was determined by single-crystal X-ray diffraction and the structures of the other phases by X-ray powder diffraction.

**Figure 2:**
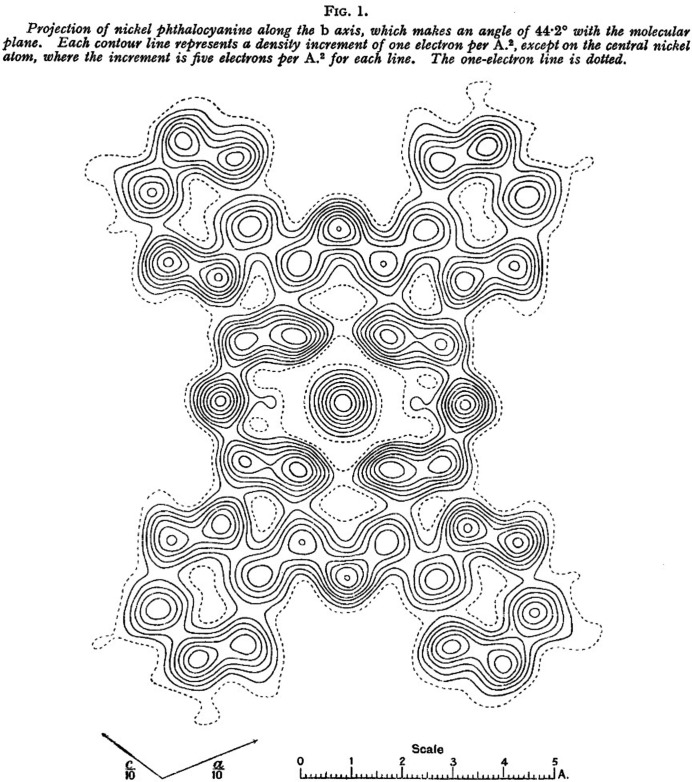
Fourier synthesis of NiPc, determined in 1937 by isomorphous replacement with metal-free Pc. The β-phase of CuPc is isostructural with this phase of NiPc. Reproduced from Robertson & Woodward (1937 ▸) with permission from the Royal Society of Chemistry.

The crystal structure of β-CuPc was determined as early as 1935–1937 by John Monteath Robertson and was among the first structures of organic copper com­plexes to be determined. Single crystals of CuPc were grown by sublimation at 580 °C and measured using moving-film cameras (Robertson, 1935 ▸). At that time, structure solution from single-crystal X-ray data was generally carried out by trial and error. Robertson observed that CuPc, NiPc and PtPc were isostructural with metal-free Pc, and solved the structures by a method which is today known as ‘isomorphic replacement’. He wrote: ‘*The usual difficulty of the unknown phase constant, which necessitates a preliminary analysis by trial, has been overcome by com­paring absolute measurements of corresponding reflections from nickel phthalo­cyanine and the metal-free compound, which leads to a direct determination of all the significant phase constants in the (h0l) zones of the two compounds, numbering about 300. A Fourier analysis of these results determines two co-ordinates [x and y] of each carbon and nitro­gen atom in the structure, and the regularity of the projection shows beyond doubt that the molecule is planar. The orientation of the molecule in the crystal is deduced, and the third co-ordinates of the atoms calculated. The results are then confirmed by a second Fourier projection of the structure, along the c axis*.’ (Robertson, 1936 ▸). Fig. 2 ▸ shows the Fourier synthesis of NiPc (Robertson & Woodward, 1937 ▸), which is isostructural with CuPc. The square-planar coordination of four-coordinated Cu atoms had not been observed before. Prior to the work of Robertson, Cu atoms had only been found in a tetrahedral geometry. Apparently, Robertson never published the atomic coordinates of CuPc, probably because the single crystals were of poorer quality than those of NiPc, PtPc and metal-free Pc.

**Figure 3:**
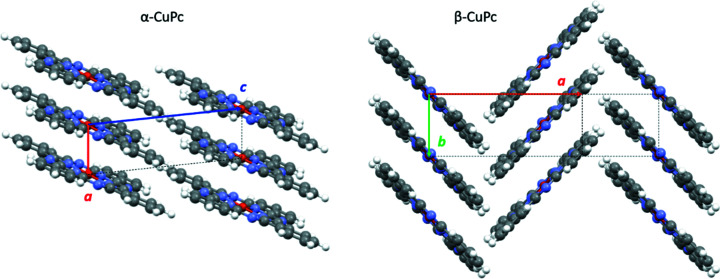
The crystal structures of α-CuPc with parallel molecular packing and β-CuPc with herringbone packing; the molecular columns run in a vertical direction. The γ- and ɛ-phases of CuPc also have herringbone structures.

The phthalo­cyanine molecule adopts an almost planar and completely conjugated structure. In all known crystal structures of CuPc, the molecules are stacked in columns. The molecular planes are not orthogonal to the column axes, but inclined, the inclination being different for different poly­morphs. Neighbouring columns are packed either in a parallel manner, giving rise to parallel molecular packing (α-phase of CuPc), or in an antiparallel mode, resulting in a herringbone packing (β-, γ- and ɛ-phases of CuPc) (Fig. 3 ▸).

For the fluorinated derivative CuPcF_16_, which is not commercially used as a pigment, two crystal structures have been reported (Yoon *et al.*, 2010 ▸; Pandey *et al.*, 2012 ▸; Jiang *et al.*, 2014 ▸). As no clear nomenclature for CuPcF_16_ poly­morphs exists, we loosely denote the Yoon structure as (I) for being published first and the Pandey structure as (II) for being published later. The structure reported by Jiang *et al.* (2014 ▸) matches phase (I) (Yoon *et al.*, 2010 ▸). The CuPcF_16_ (I) structure has a crystallographic peculiarity: it has two symmetrically independent molecules in the structure, thus forming two types of columns. Noticeably, the two types of columns have very similar inclination geometries. Neighbouring columns are arranged in an antiparallel manner, giving rise to a herringbone packing. The CuPcF_16_ (II) poly­morph is built of columns with practically the same inclination geometry as the (I) structure, but with the molecules being arranged in a parallel manner.

The fully chlorinated derivative, CuPcCl_16_, contains 16 Cl atoms. In the industrial process, chlorination is not always complete, resulting in a mixture of molecules and isomers with an average of 14–15 Cl atoms and 1–2 H atoms. The Cl deficiency does not change the crystal structure. Organic pigments are known to easily form mixed crystals (solid solution) (Schmidt *et al.*, 2006 ▸, 2007 ▸; Paulus *et al.*, 2007 ▸; Hunger & Schmidt, 2018 ▸; Schlesinger *et al.*, 2020 ▸). Correspondingly, the mixture with 14–15 Cl atoms will surely form a solid solution having the same crystal structure as CuPcCl_16_. Even the unit-cell parameters will be the same, because the statistical replacement of a few Cl atoms by H atoms does not allow the lattice to shrink. The best crystallographic model for such a structure is the description as CuPcCl_16_ with reduced occupancies of the Cl atoms.

CuPcCl_16_ (as well as CuPcCl_14–15_) is fully insoluble in all solvents, even at elevated temperature, and resists all recrystallization attempts. Consequently, single-crystal X-ray analysis is not possible. It turned out that the molecule is exceptionally stable under electron irradiation, which made it a target for high-resolution transmission electron microscopy (HR-TEM) imaging (Uyeda *et al.*, 1972 ▸, 1978 ▸; Haruta & Kurata, 2012 ▸; Yoshida *et al.*, 2015 ▸) and for electron diffraction (ED). For these purposes, thin nanocrystals were prepared on freshly cleaved (001) KCl faces by sublimation from a heated source (Uyeda *et al.*, 1972 ▸); the KCl substrate was subsequently dissolved in water.

The crystal structure of CuPcCl_16_ was solved for the first time in 1972 (Uyeda *et al.*, 1972 ▸) from an elegant combination of electron diffraction data and geometrical analysis. CuPcCl_16_ crystals were grown by sublimation on the (001) face of KCl and transferred to a TEM grid. All crystals lay flat on the TEM grid and had the same orientation along the incident electron beam. When the sample was tilted by approximately 30° away from the normal incidence, an electron diffraction zone pattern could be obtained, which was assigned as the [001] zone. The pattern showed *cmm* symmetry with the reflection condition *hk*0: *h* + *k* = 2*n*, which pointed to a *C*-centred monoclinic lattice. This pattern allowed a* and b* to be measured directly. The length of the short unit-cell vector c* was determined from needle-like crystals, which were found occasionally on the grid, allowing the [100] zone to be recorded. The monoclinic angle β* could be measured indirectly from two zonal patterns – the normal incidence pattern (at zero tilt) and the [001] pattern. The resulting unit-cell parameters were *a* = 19.62, *b* = 26.04, *c* = 3.76 Å and β = 116.5°.

The crystal structure was solved from a Patterson map generated by optical Fourier transformation: about 225 reflections from the [001] zone were manually coded into a mask, which was photographically reduced in size. Subsequently, an optical Fourier transformation was per­formed with a He–Ne laser, yielding the Patterson map. This map was compared with Patterson maps calculated for different molecular orientations. The Patterson map of the [001] zone showed a view along the molecular columns. Uyeda and co-workers assumed that at least some of the molecules were oriented approximately parallel to the basal plane of the crystal, *i.e.* orthogonal to the normal incidence direction. This assumption, which later turned out to be correct, allowed a complete structure to be built in the centrosymmetric space group *C*2/*m*, with all molecules being stacked parallel. Uyeda used a rigid molecule. The molecular position was fixed on the inversion centre. The 2/*m* site symmetry also fixes the molecular rotation around the *a* and *c* axes. Uyeda determined the molecular orientation by rotating the molecule around the *b* axis in steps of 5°. The inclination angle between the normal vector of the molecular plane and the [001] direction was determined to be 25 (5)°; hence, the molecules lay almost in the sample plane.

Although the direction of the molecular columns was fixed as [001], the exact value of the inclination angle of the molecules within the columns remain unjustified. The packing of all molecules in the sample plane, as it was proposed, was not known for any poly­morph of nonchlorinated CuPc (in α-CuPc, neighbouring molecules form steps of 0.90 to 1.61 Å). The possibility for anti­parallel packing of neighbouring columns with the formation of a herringbone structure was not excluded. A few years later, when Douglas Dorset was analysing the electron diffraction data of CuPcCl_16_, he was not speaking of a 3D structure, but conservatively focused on the single [001] zone (Dorset, 1995 ▸).

Dorset per­formed a symbolic addition (interactive phasing) of reflections in the [001] zone of CuPcCl_16_. Several strong reflections were first phased randomly. In one of the potential maps, reasonable positions of Cu and some Cl atoms were located. The positions of these atoms were then reinforced in the map and used to generate the phases for all other reflections. Once new reasonable atomic positions were found, they were added to the map. The process was repeated until no new atomic positions were found. Remaining light atoms were found in the Fourier difference map. Alternatively, the use of image phases as a basis set, supported by higher resolution electron diffraction data, allowed the resolution of the potential map to be enhanced. Being a symbol for the advances of high-resolution TEM imaging and electron diffraction in the 1990s, the CuPcCl_16_ molecule is shown on the cover of Dorset’s book ‘*Structural electron crystallography*’ (Dorset, 1995 ▸).

Thus, the structure of CuPcCl_16_, although being around for half a century, has not been crystallographically fully described and validated. Also, the space group (*C*2/*m*, *Z* = 2) remained uncertain. Even a structure in *I*2/*m* with half of the molecules shifted by *c*/2 could not be fully excluded, since in [001] projection, *C*2/*m* and *I*2/*m* cannot be distinguished. Inspired by the recent progress in the structure analysis from 3D electron diffraction (3D ED) data (Gemmi *et al.*, 2019 ▸), we decided to per­form a complete crystallographic analysis of CuPcCl_16_ by 3D ED and to place the obtained structure into the series of CuPc derivatives.

## 2. Materials and methods   

### 2.1. Growth of crystals by sublimation   

CuPcCl_16_ powder was pur­chased from Tokyo Chemical Industry and purified by train sublimation. For the TEM investigations, epitaxial CuPcCl_16_ thin crystals were prepared *via* vacuum deposition from a thermal deposition source onto a freshly cleaved (001) surface of KCl at 320 °C. The deposition rate was kept constant at 0.1 nm min^−1^. The deposition was stopped once the films reached an average thickness of 5 nm. A quartz microbalance attached to the deposition chamber monitored the weight of the deposited material. The average thickness was estimated from the weight, the deposited area and the density of the material. For most organic molecular materials, the deposited layer grows island-like rather than layer-by-layer. The island-like growth for CuPcCl_16_ was evident from measurements with an atomic force microscope (AFM). The morphology and thickness of the deposited CuPcCl_16_ crystals were estimated with an AFM (Digital Instruments NanoScope IIIa). The local crystal thickness measured by the AFM was about 50 nm. In order to improve the stability of the CuPcCl_16_ layer, a thin amorphous carbon film with a thickness of several nanometres was deposited on the top. The KCl substrate was then dissolved in water and the layer was transferred onto a gold-coated lacey carbon TEM grid. The gold precoating was used in order to improve the thermal and electric conductivity of the sample in TEM.

### 2.2. Collection of electron diffraction data in static geometry   

3D ED data were collected with a Thermo Fisher TITAN F300 TEM in nanodiffraction mode with a condenser C2 aperture of 50 µm, and an effective beam diameter on the sample of 1 µm. The data were collected at room temperature using a Fischione Advanced Tomo Holder 2020 and a Gatan US1000-XP 2k CCD camera. The *EDT-collect* program (Analitex, Stockholm, Sweden) was employed for automated data collection based on a combined stage-tilt/beam-tilt col­lection scheme. Three data sets were recorded from three different crystals for the structure analysis. The statistical characteristics of the data sets are summarized in Table S1 (see supporting information). A stage tilt increment of 3° was used for all data sets. The beam tilt increment was 0.5° for the first data set and 0.2° for the second and third data sets.

### 2.3. Collection of electron diffraction data in continuous rotation geometry   

Continuous rotation 3D ED data were collected on a Thermo Fisher TALOS TEM equipped with a fast 4k CETA camera. Electron diffraction data were collected in nanodiffraction geometry with a C2 condenser lens of 50 µm and an effective beam diameter on the sample of 1 µm. Binning 2 of the camera was used. The data were collected with a dedicated stage controlling script (see supporting information).

Two individual crystals were used for the data collection. For each crystal, several tilt series were collected within the total tilt range of ±60° with different exposure or rotation speed. For the first crystal (A), the rotation speed was kept constant (0.05 fraction of the standard speed setting) and the exposure per single frame was 0.5, 0.7 and 1 s (data sets A1, A2 and A3, respectively). These conditions resulted in effective tilt increments of 0.741, 1.043 and 1.481°, respectively. For the second crystal (B), the exposure time for a single frame was kept constant (0.5 s), while the rotation speed was varied from a 0.03 fraction of the standard speed setting to 0.1 (data sets B1–B8). The information on the continuously recorded data sets is summarized in Table S2 (supporting information).

### 2.4. Characteristic electron dose   

The characteristic electron dose, as defined in Kolb *et al.* (2010 ▸), for CuPcCl_16_ crystals was measured at room tem­per­ature at 300 kV by collecting sequences of diffraction patterns from the same position. The characteristic dose was measured to be 80 e^−^ nm^−2^. The data were collected with the total dose being below 20 e^−^ nm^−2^ per full tilt series, thus ensuring that beam damage does not lead to significant deterioration of the diffraction intensities.

### 2.5. Elemental analysis – EDX   

Energy-dispersive X-ray (EDX) spectroscopy was carried out using TALOS with a SuperX EDX detector. The spectra were quantified using the *Velox* software (Thermo Fisher). Elemental analysis resulted in an N to Cl atomic fraction ratio of 33.8:66.2, corresponding to an N:Cl ratio of 8:16, as expected. Cu-EDX could not be measured since the TEM grid contained Cu.

### 2.6. Processing of electron diffraction data   

3D ED data collected using both the combined stage-tilt/beam-tilt approach and continuous rotation were processed using the *EDT process* software (AnaliteX, Stockholm, Sweden). The frame sequences (in MRC format) of continuous rotation data sets were clipped in order to keep the un­blanked frames only, using home written MatLab scripts. An extended MRC header (Cheng *et al.*, 2015 ▸) was added to the obtained data sets, including the pixel size (scaling factor) and the tilt angle increment, calculated as described in the supporting information.

### 2.7. Merging of the ED data sets   

Separate data sets were merged using a scaling factor *f* calculated by minimizing the following *R* factor upon *f*:

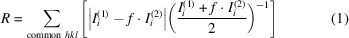

The obtained scaling factors and the metric data of the obtained data sets are listed in Table 1 ▸.

### 2.8. Structure solution analysis from electron diffraction data   

The structure was solved by direct methods as implemented in *SIR* (Burla *et al.*, 2012 ▸). All data sets – with static patterns (collected using the stage-tilt/beam-tilt combination) and by continuous rotation – produced a reasonable structure model. All structures were essentially the same. The best solution, in terms of all atoms being present in the potential map and the absence of additional peaks, was obtained from the merged data set EDT1–EDT2. The final *R* factor of the best trial model was 27.20%.

### 2.9. Structure refinement against electron diffraction data   

The structure was refined with *SHELXL* (Sheldrick, 2015 ▸) using the EDT1–EDT2 data set. Electron scattering factors were derived from Peng (1999 ▸). Model building was carried out with *shelXle* (Hübschle *et al.*, 2011 ▸). Data were merged before refinement to reduce fitting against errors in the data.

### 2.10. Powder X-ray diffraction   

A sample of commercial CuPcCl_16_ (Hostaperm Grün GNX) was obtained from Clariant (now Colourants Solutions), Frankfurt am Main, Germany. The powder was filled into a glass capillary and measured in transmission mode on a STOE Stadi-P diffractometer equipped with a Ge(111) monochromator and a Mythen 1 K detector using Cu *K*α_1_ radiation. The capillary was spun during the measurement. The pattern was measured in a 2θ range of 2–130°.

### 2.11. Fit to the powder X-ray diffraction data with deviating unit-cell parameters   

The structure determined by 3D ED was fitted to the powder data using the program *FIDEL* (Habermehl *et al.*, 2014 ▸, 2018 ▸, 2021 ▸). Different calculations were per­formed.

(1) A local fit, starting from the structure determined by ED. The fit was carried out in two space groups: (*a*) *C*2/*m*, *Z* = 2, with the molecule situated on a 2/*m* site at (0,0,0), and 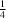 molecule per asymmetric unit; (*b*) in the subgroup *P*2_1_, *Z* = 2, with an entire molecule situated on the general position.

(2) A ‘regional fit’ starting from a large set of random structures with unit-cell parameters varying by ±2 Å, β varying by ±20° and the molecular orientation varying by a rotation of ±20° around the *b* axis. Again, two settings were investigated: (*a*) *C*2/*m*, *Z* = 2, with the molecule on a 2/*m* site; (*b*) *P*2_1_, *Z* = 2, with an entire molecule situated on the general position. In *P*2_1_, the molecule was allowed to rotate around all axes by ±20° and the molecular position was set within a range of ±0.2 in fractional coordinates in the *x*, *y* and *z* directions. In *C*2/*m*, about 25 000 starting structures were generated and in *P*2_1_ more than 100 000.

In all cases, the molecular geometry was kept fixed and a 2θ range of 3–70° was used.

### 2.12. Rietveld refinement   

Rietveld refinements were carried out with *TOPAS Academic* (Version 4.1; Coelho, 2018 ▸) using a 2θ range of 4–80°. By utilizing the *FIDEL*-to-*TOPAS* link, first an automated refinement sequence was per­formed, followed by a series of user-controlled refinements. Restraints were applied on all bond lengths and angles. The target values for the restraints were set to median values from Cambridge Structural Database (CSD) statistics provided by *MOGUL* (Bruno *et al.*, 2004 ▸; Cottrell *et al.*, 2012 ▸). A ‘flatten’ restraint was set for the entire molecule. The refinement included all atomic coordinates, unit-cell parameters, zero-point error, background (20 parameters), peak profile parameters and one overall isotropic displacement parameter.

Since mass spectroscopy of the sample used for Rietveld refinement indicated an average composition of the commercial sample of CuPcCl_14.8_ instead of CuPcCl_16_, the occupancy of the Cl atoms was set to 0.925. The peak width anisotropy was modelled with spherical harmonics of order 4. No correction for preferred orientation was applied.

### 2.13. Lattice energy minimization   

Theoretical calculations of the model CuPcCl_16_ and CuPcF_16_ crystal structures were per­formed by means of the plane-wave DFT using the Vienna Ab Initio Simulation Package (VASP, Version 5.4.4; Kresse & Hafner, 1993 ▸; Kresse & Furthmüller, 1996 ▸). The interaction between ions and electrons was described by the projector-augmented wave (PAW) method allowing an approximate all-electron wavefunction to be computed (Blöchl, 1994 ▸; Kresse & Joubert, 1999 ▸). The Generalized Gradient Approximation (GGA) under the Perdew, Burke and Ernzerhof (PBE) exchange–correlation functional (Perdew *et al.*, 1996 ▸) was applied for all calculations. A plane-wave kinetic cutoff energy of 600 eV was chosen for calculations. For the systems under consideration, having two molecules (114 atoms) per unit cell, the relative energies converged to a few µeV. Any movement of atoms was stopped if the change in the total (free) energy was smaller than 10^−5^ eV between two ionic relaxation steps (for the threshold of 10^−6^ eV set for the electronic relaxation). The many-body dispersion (MBD) energy method (MBD@rsSCS, where ‘rsSCS’ stands for range-separated self-consistent screening) of Tkatchenko and co-workers (Tkatchenko *et al.*, 2012 ▸; Ambrosetti *et al.*, 2014 ▸) was used to account for van der Waals interactions (Bučko *et al.*, 2010 ▸; Tafreshi *et al.*, 2014 ▸). The Monkhorst–Pack scheme (Monkhorst & Pack, 1976 ▸) was used for numerical integration over the Brillouin zone. A 2 × 2 × 8 Monkhorst–Pack *k*-point mesh was used for structures with parallel molecular packing, while on 8 × 2 × 2 *k*-point mesh was used for structures with herringbone packing. In both cases, the maximum number of points (8) was chosen for the direction in *k*-space associated with the shortest basis vector of the unit cell.

### 2.14. Model and data availability   

The following models were deposited in the Cambridge Structural Database (CSD):

(1) CCDC 2080221, least-squares refinement against ED data, fully occupied Cl atoms, no H atoms (*cf*. §2.9);

(2) CCDC 2080712, Rietveld refinement of 2080221 against X-ray powder data (*cf*. §2.12);

(3) CCDC 2080713, DFT optimization of 2080221 (*cf*. §2.13);

(4) CCDC 2080220, like 2080221 with H/Cl disorder (*cf*. §2.9).

## 3. Results and discussion   

### 3.1. Structure solution from 3D ED data   

**Figure 4:**
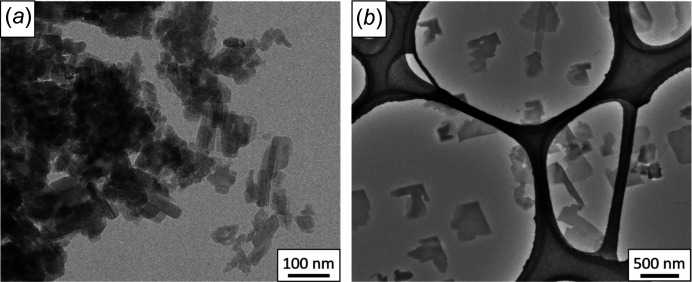
(*a*) TEM image of the original nanocrystalline CuPcCl_16_ powder and (*b*) crystals prepared through vacuum deposition for 3D ED analysis.

The commercial samples of CuPcCl_16_ were nanocrystalline powders with crystallite sizes of 20–100 nm [Fig. 4 ▸(*a*)]. The crystals were strongly agglomerated and too small for a proper 3D ED structure analysis. The crystals grown by vacuum deposition were platelets with a lateral size of approximately 0.5 µm [Fig. 4 ▸(*b*)]. Occasionally needle-like crystals were found. Despite the difference in morphology, the needle-like crystals had the same crystal structure as the platelets. The structure analysis from 3D ED data was per­formed on crystals prepared by vacuum deposition [Fig. 4 ▸(*b*)].

**Figure 5:**
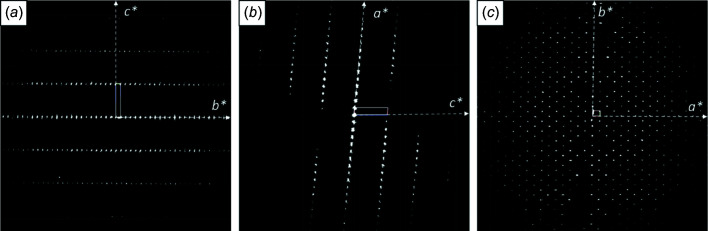
Views of 3D reconstructed reciprocal space, using data set EDT1, showing projections along (*a*) a*, (*b*) b* and (*c*) c*.

The unit cell determined from 3D ED data was monoclinic *C*-centred, with unit-cell parameters of *a* = 17.7, *b* = 25.9, *c* = 3.8 Å and β = 95.4°. No additional extinctions were detected (see Fig. 5 ▸); therefore, the space group could either be *C*2 (No. 5), *Cm* (No. 8) or *C*2/*m* (No. 12). From the unit-cell volume, it was obvious that the unit cell contains two molecules. Hence, in *C*2, the molecules must be situated in the twofold axes, in *Cm* on a mirror plane and in *C*2/*m* on a 2/*m* site. Since the molecule itself has an inversion centre, the actual symmetry is *C*2/*m* in all cases. Therefore, the space group must be *C*2/*m*.

**Figure 6:**
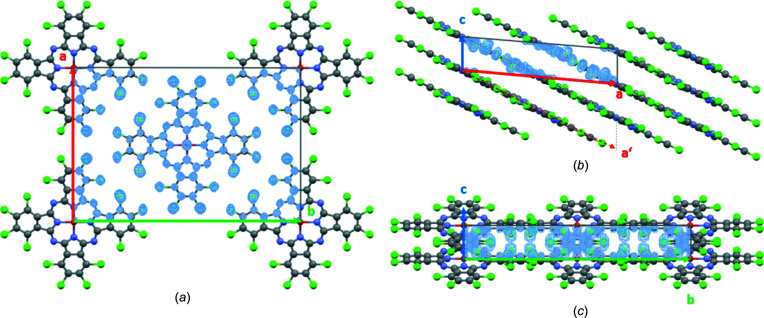
Projections of the scattering density maps obtained from direct methods (using the merged data set EDT1–EDT2) overlaid with the final structure. The view directions are (*a*) [00 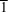 ], (*b*) [0 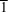 0] and (*c*) [100].

High crystal mosaicity was apparent from the reconstructed 3D data [Figs. 5 ▸(*a*) and 5 ▸(*b*), and Fig. S3]. As the data reduction software used takes the maximum intensity of each reflection, the mosaicity did not influence the structure solution. The structure was solved by direct methods. The best density map with the lowest residual peaks was obtained from the merged EDT1–EDT2 data set. One can see that all atoms of the phthalo­cyanine molecule were clearly detected in the electron-density map (Fig. 6 ▸). Some atomic species (C and N) were not assigned properly and had to be corrected *posteriori*.

It should be noted that all data sets – with static patterns (recorded using the combined stage-tilt/beam-tilt collection scheme) and continuous rotation patterns – per­formed well for structure solution. They also showed similar statistics in terms of completeness and *R*_int_ factors.

### 3.2. Refinement against ED data   

The structure was refined kinematically in *SHELXL* (Sheldrick, 2015 ▸).

As a test, we modelled a Cl/H disorder for each of the four symmetrically independent Cl atoms and refined the occupancy of each Cl atom independently. During this step, we restrained the structure to be flat. In the absence of such a restraint, the H atoms would deviate from the plane in an unrealistic manner, possibly due to the incompleteness of the dataset. Occupancies and *U* values were strongly correlated (correlation coefficients of >0.77 between Occ and *U*_iso_), and the resulting occupancies depended on the starting values (see Tables S3 and S4). The refinement giving the best fit and the lowest *R* values led to occupancies of 1.17 (5), 0.97 (5), 1.21 (5) and 1.36 (5) for an isotropic refinement, and to occupancies of 1.29 (5), 1.08 (5), 1.10 (5) and 1.27 (5) for an anisotropic refinement. Hence, all Cl atoms were fully occupied within the limits of the method and the final refinement was per­formed without disorder, *i.e.* with fully occupied Cl atoms. The structure was refined anisotropically without restraints, except for the RIGU instruction (rigid-body restraints) (Thorn *et al.*, 2012 ▸). This resulted in *R*1 = 28.17% for all 1796 reflections and *R*1 = 26.52% for 1505 reflections with *I* > 2σ(*I*).

Dynamical scattering is unavoidably present in ED data (Dorset *et al.*, 1992 ▸). The presence of multiple scattering in CuPcCl_16_ ED patterns was already noticed by Dorset (1995 ▸). Recently, software allowing for the dynamical refinement of 3D ED data was developed (Palatinus *et al.*, 2019 ▸). Thus, as a next step, dynamical refinement (Palatinus *et al.*, 2015 ▸; Brázda *et al.*, 2019 ▸; Debost *et al.*, 2020 ▸) was attempted. One of the key parameters of dynamical refinement is the effective crystal thickness at a given crystallographic orientation. It turned out that CuPcCl_16_ crystals systematically showed a very high mosaicity (see Fig. S3), which made the determination of the thickness unreliable and the whole dynamical refinement procedure unstable.

### 3.3. Refinement of unit-cell parameters using powder X-ray diffraction   

The unit-cell parameters determined from 3D ED were subsequently refined by a fit to powder X-ray diffraction (PXRD) data.

**Figure 7:**
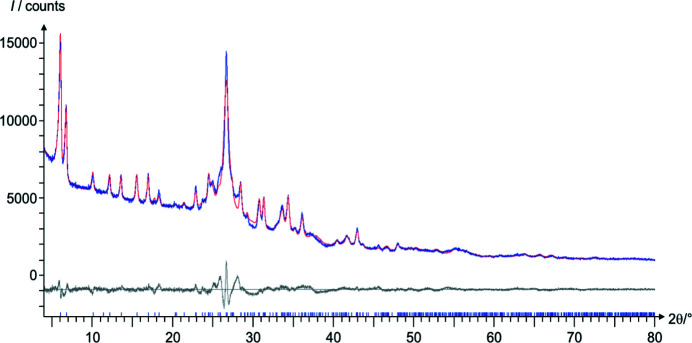
Rietveld fit of the 3D ED structure against PXRD data. Experimentally measured data are shown in blue, the simulated plot is in red and the difference curve is in grey. The blue tick marks denote the reflection positions.

CuPcCl_16_ is a nanocrystalline powder. Correspondingly, the reflections in the PXRD pattern are broad and, despite being very accurately measured, the powder data are of limited quality (Fig. 7 ▸). The powder pattern could not be indexed *ab* *initio* in any reliable way. During indexing with the ED cell parameters, an additional difficulty was faced, *i.e.* most of the visible reflections belonged to the *hk*0 zone. The only strong reflection with *l* ≠ 0 was 201 at 2θ = 26.70°. All other reflections with *l* ≠ 0 were either weak or overlapping with *hk*0 reflections. Hence, a* and b* could be easily and reliably determined, but information on c* and β* was rather limited. In particular, the angle β* was ill-defined.

The powder pattern, simulated from the 3D ED structure exhibited a similar peak intensity pattern as the experimental PXRD data, but the peaks appeared shifted, thereby making the Rietveld refinement difficult. Hence, the program *FIDEL* (Fit with Deviating Lattice parameters; Habermehl *et al.*, 2014 ▸) was used to adjust the unit-cell parameters before per­forming a Rietveld refinement. *FIDEL* can fit a trial structure to a powder pattern, even if the unit-cell parameters deviate significantly. In contrast to the point-by-point com­parison of experimental and simulated patterns in the Rietveld method, *FIDEL* uses a similarity measure *S*_12_ based on cross-correlation functions, which also includes neighbouring data points within a user-defined 2θ range (Habermehl *et al.*, 2014 ▸). For CuPcCl_16_, a neighbourhood 2θ range of 0.5° in the raw fit and 0.1° in the fine fit was used. Starting from the 3D ED structure, the unit-cell parameters and molecular orientation were refined, resulting in quite a good match to the experimental powder data. Subsequently, a Rietveld refinement (Loopstra & Rietveld, 1969 ▸) was per­formed, with restraints on all bond lengths, bond angles and molecular planarity. After about 30 consecutive runs with varying conditions and parametrizations, an acceptable fit could be achieved (see Fig. 7 ▸). During the *FIDEL* fit and the Rietveld refinement, the angle β changed from 95.05 *via* 95.84 to 95.34°. The final crystallographic data are given in Table 2 ▸ and Table S3.

### 3.4. Attempts at a crystal structure solution from powder X-ray diffraction data   

Every structure refinement procedure, including a Rietveld refinement, is not a global optimization, but a local optimization, which starts from a given structural model and searches for the optimal fit to the experimental data in the vicinity of the starting structure. If the starting structure is too far off, the refinement may end in a local minimum, which may give an acceptable fit to the data, but with a completely wrong crystal structure (see, for example, Buchsbaum & Schmidt, 2007 ▸). In order to ensure that the Rietveld refinement leads to the global minimum, we started with the global optimization method of *FIDEL* (Habermehl *et al.*, 2018 ▸, 2021 ▸). The global fit approach of *FIDEL* is designed to search for structures matching the powder data, by exploring a complete parameter space, including unit-cell parameters, molecular position and orientation, in a given space group. This is carried out by generating a large number of random trial structures (within the parameter space), which are subsequently fitted to the powder pattern by a hierarchical procedure. The similarity measure *S*_12_ is used for the com­parison and fitting of the structural models to the experimental data.

For CuPcCl_16_, the method was used in the form of a ‘regional fit’, *i.e.* exploring only the parameter space region around the structure determined by ED. The random starting values for the unit-cell parameters *a*, *b* and *c* were allowed to vary by ±2 Å each, the angle β by ±20° and the molecular orientation by ±20°. The regional fit was carried out twice, in *C*2/*m*, *Z* = 2, with the molecule fixed at the 2/*m* site, and in its subgroup *P*2_1_, *Z* = 2, with an entire molecule on the general position.

Both fits lead to similar results: the structural model derived from ED could be validated as a very good match to the powder data. However, it did not represent a unique solution. Alarmingly, both regional fits revealed an alternative structural model with a β angle of 99° instead of 95°, that matched the powder data even slightly better. In the subsequent initial Rietveld refinement, this structure gave an acceptable fit with *R* values only slightly worse than for the correct structure. However, during the following careful Rietveld refinements, the β-angle changed from 99.3 *via* 97.7 to 95.3°, and the structure became identical to the first structure. Thus, the structure shown in Table 2 ▸ is indeed that which gives the best fit to the powder X-ray diffraction data.

### 3.5. Structure validation by DFT   

Finally, we validated the crystal structure using lattice energy minimization, as was done previously for other molecular compounds solved from 3D ED data (Gorelik *et al.*, 2012 ▸).

Upon DFT energy minimization, the ED structure remained stable and the unit-cell parameters of the minimized structure were *a* = 17.7328, *b* = 26.1583, *c* = 3.8418 Å and β = 95.048° (see Table 2 ▸). The average atomic displacement was 0.075 Å, which is way below the threshold of 0.25 Å established by van de Streek & Neumann (2010 ▸), meaning that the structure is correct.

The obtained energy of the crystal structure ( 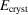 ) was −794.806 eV. From this value, the lattice packing energy ( 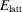 ) could be calculated using the relation

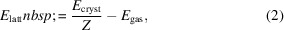

where 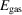 corresponds to the energy of one isolated molecule in the lowest-energy gas-phase conformation and *Z* = 2 is the number of molecules in the unit cell. The obtained 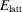 for the CuPcCl_16_ structure was −3.109 eV.

### 3.6. Crystal structure of CuPcCl_16_   

As in the structure of Uyeda *et al.* (1972 ▸), the molecules create a *cmm* pattern when viewed along the column direction [*c* column, Fig. 6 ▸(*a*)]. The molecules are packed in a parallel manner [Fig. 6 ▸(*b*)], best seen along the *b* axis. Moreover, the molecules form perfect layers parallel to (201). In the unit vector settings proposed by Uyeda, the basis vector ***a*** was selected along the molecular plane, denoted as the ***a*′** vector in Fig. 6 ▸(*b*). The transformation of the Uyeda basis to the standard settings can be carried out using the matrix (1 0 2; 0 1 0; 0 0 1). The obtained unit-cell metric matches well the unit cell determined from 3D ED (see Table 2 ▸). The atomic positions match quite well, too. Hence, the proposed crystal structure of Uyeda *et al.* (1972 ▸) can be confirmed.

### 3.7. Comparison with crystal structures of other CuPc derivatives   

All known CuPc structures consist of planar molecules packed into columns with an approximate distance of 3.4 Å between the molecular planes. The molecular planes are inclined with respect to the column axis and the columns are packed into a crystal structure.

#### 3.7.1. Arrangement of molecules in the columns   

The mutual shift of the planar molecules within molecular columns can be described phenomenologically using different pairs of geometric parameters – ‘pitch and roll’ inclinations (Curtis *et al.*, 2004 ▸) or direction cosines of the shift vector (Milita *et al.*, 2020 ▸).

**Figure 8:**
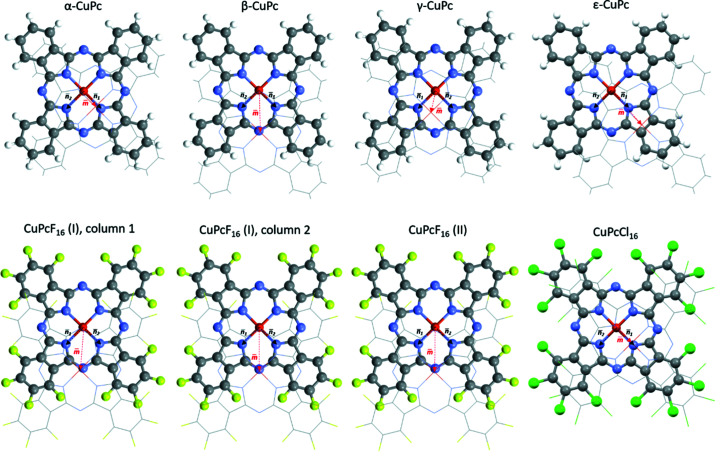
Relative shift of the CuPc molecules within columns, with the view orthogonal to the molecule plane. The top row contains the known structures of CuPc poly­morphs and bottom row contains the CuPcF_16_ and newly determined CuPcCl_16_ structures. In each case, the top molecules are drawn in wireframe for clarity.

The inclination of the molecular plane with respect to the stacking column direction, present in all Pc poly­morphs, originates from the mutual shift of the neighbouring molecules in a column. In order to describe the shift geometrically, we define two orthogonal vectors within a molecule plane ***n*_1_** and ***n*_2_**, along the two orthogonal Cu—N coordination bonds, and a vector between the copper positions in neighbouring layers as vector ***m*** (Fig. 8 ▸). The shift of the neighbouring molecules can also be described using the two angles **φ_1_** = 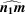 and **φ_2_** = 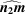 . The values of ***n*_1_**, ***n*_2_**, **φ_1_**, **φ_2_** and |**m**| are summarized in Table 3 ▸. Due to the molecular symmetry, the vectors ***n*_1_** and ***n*_2_** are interchangeable. We always select ***n*_1_** as being closer to ***m***.

**Figure 9:**
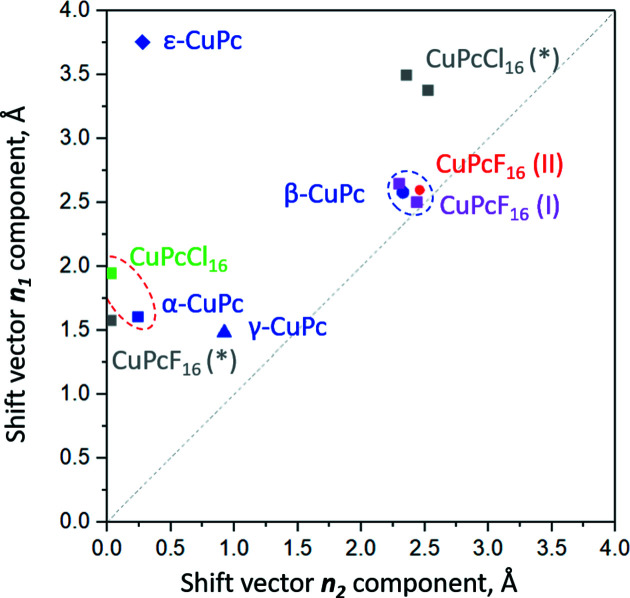
The molecular packing within columns for different structures represented as a map of lateral shift vectors in ***n*_1_ *n*_2_** basis. CuPcF_16_ (I) contains two symmetry-independent molecules. The asterisks (*) denote hypothetical structures. The grey dashed line is a plot diagonal with ***n*_1_** = ***n*_2_**.

From Fig. 9 ▸ and from the values given in Table 3 ▸, one can see that the mutual shifts of neighbouring molecules are different for all CuPc poly­morphs. In β-CuPc, the molecules are shifted essentially along a diagonal direction (**φ_1_** 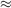 **φ_2_** and ***n*_1_** 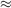 ***n*_2_**). In ɛ-CuPc, the molecular shift occurs almost along a Cu—N coordination bond (**φ_2_** is close to 90° and ***n*_2_** is close to 0).

In α-CuPc and β-CuPc, the molecules are shifted in such a way that the Cu atom lies right above different N atoms of an adjacent molecule (Fig. 8 ▸). In γ-CuPc, the Cu atom appears in the middle of three N atoms of the neighbouring layer. No clear geometrical relation between the Cu and N atoms of adjacent layers could be identified in ɛ-CuPc. In all poly­morphs, the distance between the Cu atom and N atoms of a neighbouring molecule is always greater than 3.3 Å. As a result, this contact is not a coordination bond of the Cu atom, but a van der Waals interaction, supported by electrostatic interaction between the positively charged Cu atom and the negatively charged N atom.

In the newly determined structure of CuPcCl_16_, the molecules are shifted along one of the Cu—N coordination bonds (**φ_2_** = 90° and ***n*_2_** = 0), so that the Cu atom lies in the vicinity of an N atom of a neighbouring molecule, as in α-CuPc.

It is interesting to compare the mutual shift geometry of neighbouring molecules for different poly­morphs. Here we decided to use the lengths of the ***n*_1_** and ***n*_2_** vector. Fig. 9 ▸ shows a graphical representation of the lateral shift vectors from Table 3 ▸. CuPc poly­morphs (shown in blue) are scattered over the map, giving rise to four different molecular packings. The stacking of CuPcF_16_ molecules in both known poly­morphs mimics the β-phase of CuPc, although phase I of CuPcF_16_ exhibits a herringbone packing and phase II a parallel packing. Obviously, the diagonal molecular shift in β-CuPc, with the Cu atom lying above an N atom, forms a very attractive geometry also for CuPcF_16_. Thus, the molecular stacking of β-CuPc, CuPcF_16_ (I) and CuPcF_16_ (II) form one group, highlighted by a blue dashed ellipse in Fig. 9 ▸. Noticeably, the CuPcCl_16_ molecules follow the geometry of the stacking of α-CuPc, with a shift along a Cu—N coordination bond, thus forming a different molecular stacking group, marked by a red dashed ellipse (Fig. 9 ▸).

#### 3.7.2. Packing of the columns   

In the crystal structures of CuPc and derivatives, the molecular columns are packed either with the parallel inclination forming a parallel packing or with an antiparallel orientation of the columns resulting in a herringbone structure. In the herringbone structures, the angle between the molecules of neighbouring columns is given by the molecular inclination within the columns and the rotation of the columns with respect to each other. These values range from 28.1° for a relatively ‘flat’ CuPcF_16_ (I) structure to being almost orthogonal in β-CuPc (see Table 3 ▸).

**Figure 10:**
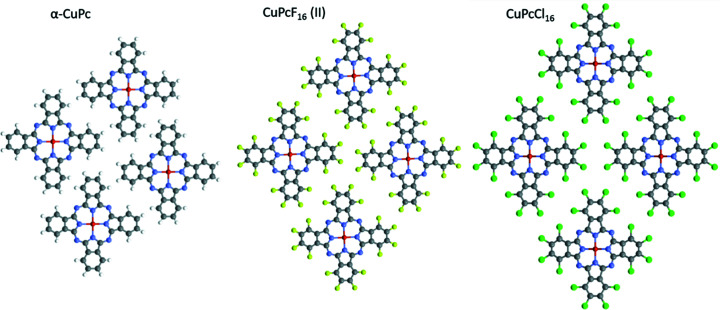
The molecular plane for parallel packing structures of α-CuPc, CuPcF_16_ (II) and CuPcCl_16_.

In the crystal structures with parallel packing [α-CuPc, CuPcF_16_ (II), CuPcCl_16_], a layer segment containing four molecules was extracted (see Fig. 10 ▸). In α-CuPc and CuPcF_16_ (II), the layer is not perfectly planar; the molecules are shifted in height from the virtual centroid plane of the layer by 1.3, 0.2, −0.2 and −1.3 Å for α-CuPc, and by −0.3, 0, 0 and 0.3 Å for CuPcF_16_ (II). In the CuPcCl_16_ structure, the molecules form an ideal plane. From Fig. 10 ▸ one can see that the molecules form a windmill pattern for the hydrogenated molecule; the pattern is less pronounced for the fluorinated molecule and turns into a *cmm* pattern for the chlorinated molecule. The windmill rotation is likely associated with the small size of H atoms and disappears in the series H →F→Cl, as Cl atoms occupy all available space between the molecules. Correspondingly, the crystal symmetry increases from *P* 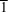 , *Z* = 1, for α-CuPc and CuPcF_16_ (II), to *C*2/*m*, *Z* = 2, for CuPcCl_16_.

### 3.8. Lattice energy calculations of real and hypothetical poly­morphs   

Having spotted all similarities and discrepancies between the structures we arrived at the question of what are the thermodynamic stabilities of different packings and which other packings would be possible. A proper answer to these questions can only be given by an extensive crystal structure prediction (CSP) study (Price & Brandenburg, 2017 ▸). The CSP is a highly demanding task and is usually per­formed for molecules of pharmaceutical importance. Pc is a rigid molecule and should not present a strong challenge for CSP, yet we are not aware of any reports of CSP for Pc molecules of any composition. We hope that this article will attract the interest of computational chemists and that a pool of predicted crystal packing will be available soon.

It is interesting to see whether the parallel packing of CuPcCl_16_ with the Cu atom above the N atom would also be possible for CuPcF_16_ molecules, and the other way around, if a crystal structure of CuPcCl_16_ with herringbone packing like in CuPcF_16_ (I) would be stable. Therefore, we per­formed lattice energy minimizations for these hypothetical structures, using the DFT+MBD method.

First, in the crystal structure of CuPcCl_16_, all the Cl atoms were substituted by F. In this way, we produced a hypothetical CuPcF_16_ (*) structure with parallel molecular packing in *C*2/*m*. After lattice energy minimization, the unit-cell parameters were *a* = 16.2747, *b* = 23.8354, *c* = 3.6461 Å, β = 94.363° and *V* = 1410.289 Å^3^. The parallel packing of the molecules was preserved. The stacking of the molecules within a single column can be described by **φ_1_** = 64.3° and **φ_2_** = 90°, and lateral shifts of ***n*_1_** = 1.58 Å and ***n*_2_** = 0 Å (see Fig. 9 ▸). Due to the smaller diameter of F atoms, the molecules move closer within a layer (Fig. S4).

Next, a hypothetical herringbone structure of CuPcCl_16_ was constructed from the crystal structure of CuPcF_16_ (I) by exchanging F atoms by Cl. After a subsequent optimization, the hypothetical CuPcCl_16_ (*) structure had unit-cell parameters of *a* = 5.3598, *b* = 11.6114, *c* = 29.5560 Å, α = 86.643, β = 88.508, γ = 85.713° and *V* = 1830.672 Å^3^. The two crystallographically independent molecular columns showed geometries with **φ_1_** = 50.95°, **φ_2_** = 61.90°, ***n*_1_** = 3.38 Å and ***n*_2_** = 2.52 Å and **φ_1_** = 49.27°, **φ_2_** = 63.99°, ***n*_1_** = 3.50 Å and ***n*_2_** = 2.35 Å (see Fig. 9 ▸).

The lattice packing energies *E*_latt_ of these structures revealed that the experimentally observed parallel packing of CuPcCl_16_ is 0.175 eV more stable than for the hypothetical herringbone structure CuPcCl_16_ (*), built from CuPcF_16_ (II). Interestingly, the molecular packing of CuPcF_16_ resembling the parallel packing of CuPcCl_16_ is 0.204 eV less stable than its herringbone packing. The parallel packing of CuPcF_16_ (II) has almost the same lattice energy as its poly­morph CuPcF_16_ (I); the lattice energy is in favour of phase (II) by 0.001 eV.

A typical energy gap between the thermodynamically most stable poly­morph and a metastable, but still experimentally observed, crystal form is about 3 kcal mol^−1^ (0.13 eV) (Neumann & van de Streek, 2018 ▸). Calculated values, which are above 0.13 eV, indicate that a structure like CuPcCl_16_ is unlikely to be observed for CuPcF_16_. Similarly, a herringbone packing as in CuPcF_16_ (I) will probably not be realized for the CuPcCl_16_ molecule. This conclusion agrees with the experimental observation that (to the best of our knowledge) no other poly­morphic form of CuPcCl_16_ has ever been detected.

## 4. Conclusions   

The CuPcCl_16_ crystal structure was solved from nanocrystals grown by vacuum deposition using 3D electron diffraction data. 3D ED data consisting of static patterns (collected through a combined stage-tilt/beam-tilt scheme) and continuous rotation data showed comparable quality in terms of structure solution. The structure was solved by direct methods and subsequently kinematically refined against ED data. In the crystal structure, the molecules are arranged in parallel planar layers. The structure matches the model proposed by Uyeda *et al.* (1972 ▸). The obtained crystal structure agreed with the results of a Rietveld refinement from powder X-ray diffraction data of a nanocrystalline commercial sample of CuPcCl_16_. Finally, the crystal structure was confirmed by DFT calculations, and lattice–energy minimizations explained why the crystal structure of CuPcCl_16_ is different from both poly­morphs of CuPcF_16_.

## Supplementary Material

Crystal structure: contains datablock(s) CuPcCl16_ED_anisoADP, CuPcCl16_ED_final, CuPcCl16_from_Rietveld, CuPcCl16_VASP_PBE-MBD_2x2x8_600eV. DOI: 10.1107/S2052520621006806/je5043sup1.cif

Structure factors: contains datablock(s) 2080220. DOI: 10.1107/S2052520621006806/je5043Isup2.hkl

cif file of the structure from Rietveld refinement. DOI: 10.1107/S2052520621006806/je5043sup3.txt

Structure factors: contains datablock(s) CuPcCl16_ED_final. DOI: 10.1107/S2052520621006806/je5043CuPcCl16_ED_finalsup4.hkl

Structure factors: contains datablock(s) CuPcCl16_ED_anisoADP. DOI: 10.1107/S2052520621006806/je5043CuPcCl16_ED_anisoADPsup5.hkl

Rietveld powder data: contains datablock(s) CuPcCl16_from_Rietveld. DOI: 10.1107/S2052520621006806/je5043CuPcCl16_from_Rietveldsup6.rtv

Technical data for 3D ED, kinematical refinement and powder X-ray refinement. DOI: 10.1107/S2052520621006806/je5043sup7.pdf

CCDC references: 2080220, 2080221, 2080712, 2080713

## Figures and Tables

**Table 1:** Merged 3D ED data sets used for structure analysis

Data set name	EDT1–EDT2	EDT1–EDT3	EDT2–EDT3	EDT1–EDT2–EDT3	A1–B3
Scaling factors	1:4.8	1:5.1	1:1.3	1:4.8:5.1	1.9:1
*N* independent reflections	1395	1292	1135	1479	1380
*R*_int_(*F*) (%)	18.41	23.86	16.43	25.85	17.07
Completeness (%) (0.8 Å)	76	70	65	81	75

**Table 2:** Crystal data and structure refinement parameters of CuPcCl_16_

	3D ED	Rietveld refinement (PXRD)	DFT calculations	Uyeda *et al.* (1972 ▸)
Empirical formula	C_32_Cl_16_CuN_8_			
Molecular weight (g mol^−1^)	1127.15			
Crystal system	Monoclinic			
Space group	*C*2/*m*			
*a* (Å)	17.7	17.5447 (17)	17.7328	17.60
*b* (Å)	25.9	25.986 (2)	26.1583	26.08
*c* (Å)	3.8	3.7631 (6)	3.8418	3.76
β (°)	95.4	95.336 (16)	95.048	94.02
*V* (Å^3^)	1734.3	1708.2 (3)	1775.147	1721.8
*Z*	2			
ρ_calc_ (g cm^−1^)	2.158	2.191	2.109	2.174
Goniometer tilt range (°)	−60…48	2θ = 3–80		Individual zone patterns
No. of measured reflns	7998	546		
No. of independent reflns/*R*_int_ (%)	1796, 30.6			*ca* 190 (*hk*0 reflections within the resolution limit of 1 Å)
Confidence values (%)	*R*1 = 27.38	*R*_p_ = 2.679		No refinement performed
		*R*_wp_ = 3.701		
		*R*_Bragg_ = 1.113		
GOF		2.079		

**Table 3:** Geometrical parameters (see text) of the shift of molecules within molecular columns for different CuPc poly­morphs, CuPcF_16_ poly­morphs and CuPcCl_16_

Crystal structure	**φ_1_** and **φ_2_** (°)	| ***m*** | (Å)	Lateral shift vector along ***n*_1_** and ***n*_2_** (Å)	Molecular packing in the crystal structure, angle (°) between molecular planes of neighbouring columns
α-CuPc	65.0, 86.4	3.805	1.61, 0.24	Parallel
β-CuPc	57.5, 61.0	4.801	2.58, 2.33	Herringbone, 89.4
γ-CuPc	67.1, 76.0	3.813	1.48, 0.92	Herringbone, 54.8
ɛ-CuPc	41.3, 86.8	5.000	3.76, 0.28	Herringbone, 53.5
CuPcF_16_ (I)	56.5, 61.4	4.796	2.65, 2.30	Herringbone, 28.1
	58.5, 59.5	4.796	2.51, 2.43	
CuPcF_16_ (II)	57.7, 59.6	4.860	2.60, 2.46	Parallel
CuPcCl_16_	59.4, 90	3.833	1.95, 0	Parallel

## Footnotes

1 We used Matrix-Assisted Laser Desorption/Ionization (MALDI). In this technique, a molecule is embedded in a matrix, which is subsequently evaporated by a laser, so that the molecule is transferred to the gas phase, and analysed by mass spectrometry (MS). In the case of an organic pigment, the matrix is not necessary and the analysis can be carried out directly from the printed paper. Since the pigment absorbs the laser beam much more strongly than the rest of the paper, the gas phase contains mainly the pigment molecules, which can then be analysed by MS. In this way, we found CuPc using a small piece of a printed edition of *Acta Crystallographica Section B*. Of course, MS cannot distinguish between poly­morphs. However, it is known that normally the β-phase is used for the four-colour printing scheme (cyan, magenta, yellow, black) because it matches the required cyan shade. The commercial α-phase is usually a solid solution of CuPc with monochloro-CuPc (which would have been visible in the MS). The ɛ-phase is much more reddish and hence unsuitable for cyan shades. In principle, a cyan shade could be obtained by mixing blue with green or yellow, but that would be more complicated and more expensive than simply using β-CuPc. Thus, we are quite sure that *Acta Crystallographica* is indeed printed with the β-phase of CuPc.
