# Supplementary material for: Crystal structure of copper perchlorophthalo­cyanine analysed by 3D electron diffraction
Source: Acta Crystallogr B Struct Sci Cryst Eng Mater. 2021 Jul 29;77(Pt 4):662–75. doi: 10.1107/S2052520621006806 (PMC8337017; doi:10.1107/S2052520621006806)
Supplement: Supplementary file 7 [file b-77-00662-sup7.pdf]

STRUCTURAL SCIENCE
CRYSTAL ENGINEERING
MATERIALS

Volume 77 (2021)

Supporting information for article:

Crystal structure of copper perchlorophthalocyanine analysed by 3D electron diffraction

Tatiana E. Gorelik, Stefan Habermehl, Aleksandr A. Shubin, Tim Gruene, Kaname Yoshida, Peter Oleynikov, Ute Kaiser and Martin Schmidt

Contents

Continuous electron diffraction data collection 1

Technical data of all collected 3D ED datasets 2

On electron dose distribution during 3D ED data collection static patterns vs continuous rotation 3

Kinematical refinement against 3D ED data 4

Dynamical refinement against 3D ED data 5

FIDEL fits 6

DFT calculations 8

Continuous electron diffraction data collection

A JavaScript controlling the goniometer speed was written. The script code is available at https://github.com/EvgenyGorelik/continuous_rotation. The script is performing the following steps: (i) a short delay is built in in order to give the operator a possibility to switch on the camera recording manually; (ii) then the beam is un-blanked and the goniometer starts rotating with a given speed to the final destination; (iii) as the final destination is reached, the beam is blanked by the script. Finally, the operator can switch off the camera recording. The data is stored in MRC file format with a few blanked frames at the beginning of the data set and at the end. The number of un-blanked frames N defines the effective tilt increment as following: $(\alpha_{end}-\alpha_{beg})/N$. A screenshot of the script interface is shown in Figure S1.

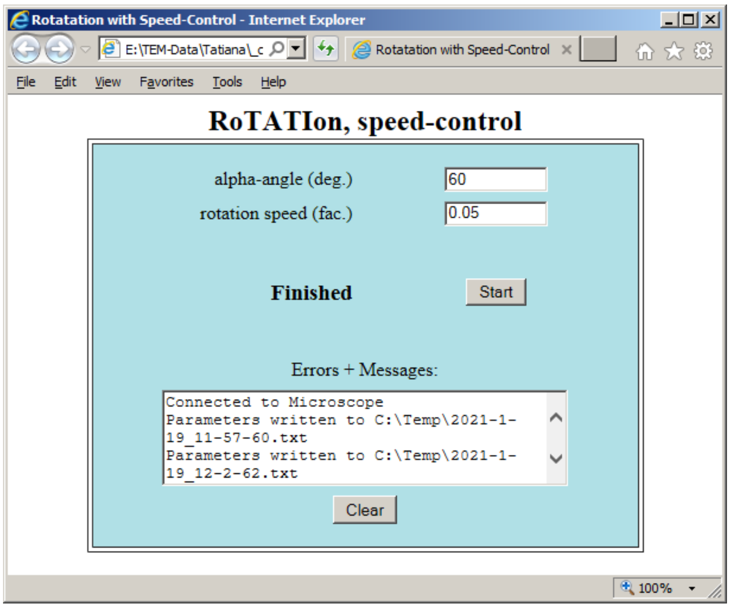

Figure S1 Screen shot of the goniometer controlling script.

Technical data of all collected 3D ED datasets

Table S1 EDT data sets with static patterns, recorded through a combined stage tilt / beam tilt acquisition strategy.

Dataset name	EDT-1	EDT-2	EDT-3
Total tilt range	-60 … 45	-60 … 48	-61 … 59
Stage tilt increment	3°	3°	3°
Beam tilt increment	0.5°	0.2°	0.2°
	37 / 6	38 / 15	42 /15
Unit cell parameters	17.7 Å, 25.9 Å, 3.8 Å, 89.2°, 95.3°, 91.1°	16.9 Å, 25.4 Å, 3.7 Å, 90.8°, 95.1°, 89.2°	17.1 Å, 25.2 Å, 3.9 Å, 89.6°, 95.8°, 89.9°
N independent reflections	1142	1139	1077
Rint(F)	11.73%	15.36%	12.83%
Completeness (0.8 Å)	62 %	65 %	59 %

Table S2 Continuous rotation datasets.

Dataset name	A1	A2	A3
Total tilt range	-60 … 60	-60 … 60	-60 … 60
Exposure per frame, s	0.5	0.7	1
Rotation speed factor	0.05	0.05	0.05
Effective tilt increment	0.741°	1.043°	1.481°
Unit cell	17.7 Å, 26.5 Å, 3.9 Å<br>89.6°, 96.2°, 90.0°	17.9 Å, 26.7 Å, 3.8 Å, 89.9°, β=96.5°, 89.9°	17.7 Å, 26.4 Å, 3.8, Å<br>90.1°, 95.4°, 89.4°
N independent reflections	1240	1121	1248
Rint(F)	17.16 %	19.49 %	18.58 %
Completeness (0.8 Å)	68 %	61 %	68 %

Dataset name	B1	B2	B3
Total tilt range	-60 … 60	-60 … 60	-60 … 60
Exposure per frame, s	0.5	0.5	0.5
Rotation speed factor	0.03	0.04	0.05
Effective tilt increment	0.446°	0.594°	0.741°
Unit cell	17.7 Å, 26.4 Å, 3.8, Å 89.3°, 95.3°, 90.6°	17.7 Å, 26.4 Å, 3.8 Å, 89.4°, 95.4°, 90.4°	17.7 Å, 24.7 Å, 3.8 Å, 91.1°, 95.3°, 89.6°
N independent reflections	1304	1349	1330

Rint(F)	14.71 %	14.43 %	14.10 %
Completeness (0.8 Å)	71 %	73 %	72 %

Dataset name	B4	B5	B6
Total tilt range	-60 … 60	-60 … 60	-60 … 60
Exposure per frame, s	0.5	0.5	0.5
Rotation speed factor	0.06	0.07	0.08
Effective tilt increment	0.888°	1.034°	1.188°
Unit cell	17.7 Å, 26.4 Å, 3.8 Å, 90.6°, 95.2°, 89.6°	17.7 Å, 26.4 Å, 3.8 Å, 90.6°, 95.0°, 89.6°	17.7 Å, 26.4 Å, 3.8 Å, 90.7°, 95.3°, 89.7°
N independent reflections	1314	1287	1269
Rint(F)	13.80 %	13.84 %	13.64 %
Completeness (0.8 Å)	73 %	70 %	69 %

Dataset name	B7	B8
Total tilt range	-60 … 60	-60 … 60
Exposure per frame, s	0.5	0.5
Rotation speed factor	0.09	0.1
Effective tilt increment	1.318°	1.481°
Unit cell	17.7 Å, 26.4 Å, 3.9 Å, 89.5°, 94.6°, 90.4°	17.7 Å, 26.4 Å, 3.8 Å, 89.5°, 95.2°, 90.1°
N independent reflections	1256	1229
Rint(F)	13.79 %	14.06 %
Completeness (0.8 Å)	68 %	67 %

On electron dose distribution during 3D ED data collection static patterns vs continuous rotation

Continuous rotation patterns can be seen as “linear” precession patterns. Different geometry of data collection may need an appropriate Lorentz correction. In this work we did not use any correction scheme.

The difference between static patters and continuous rotation may become crucial for beam sensitive crystals. $CuPcCl_{16}$ crystals were relatively stable, we did not have to make any significant precautions during the data collection. For static patterns, the amount of electron dose received by a crystal is proportional to the number of frames, which is, in turn, related to the total tilt range collected and the

tilt increment, and the exposure time per frame. In our case, the largest tilt increment used was 0.5°, the exposure time per frame was 0.5 s.

For continuous rotation, the number of frames is inversely related to the effective tilt increment, which is determined by a combination of exposure and the rotation speed. In Figure 7 different values of effective tilt increment are plotted against the speed settings, spots on the red dashed line were obtained using 0.5 s exposure, on the green – 0.5s, on the blue line 1 s. The total electron dose received by the crystal using the selected parameters is schematically represented by the size of the blue spots. We did not see any quality worsening for data sets recorded with high effective tilt increment. Therefore, the combination of high speed and low exposure will determine the strategy for beam sensitive materials. An obvious disadvantage of continuous rotation data acquisition is the absence of crystal tracking during the data collection, which sets high demands on the goniometer quality and limits on crystal size.

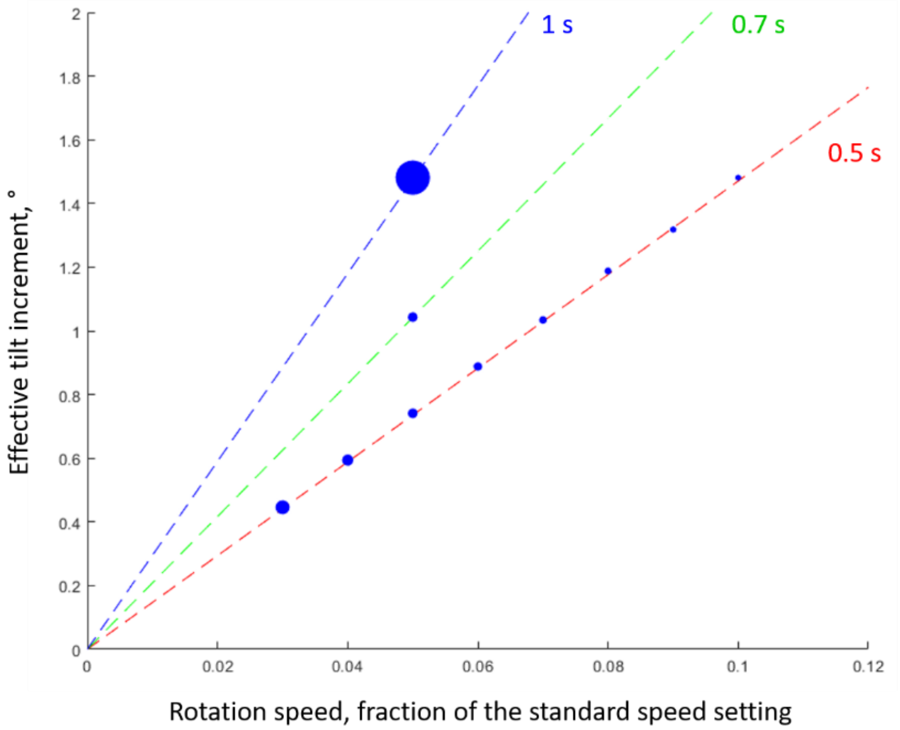

Figure S2 Interplay of parameters for continuous rotation as used in our experimental settings.

Kinematical refinement against 3D ED data

Table S3. Occupancies for each Cl-atom, anisotropic refinement, 108 RIGU restraints, 137 parameters.

Start occupancy	End occupancy				R1
	Cl1	Cl2	Cl3	Cl4	
0.1	0.87	0.69	0.90	1.00	28.43
0.5	.86	69	.91	1.01	28.49
0.6	.86	.67	.91	1.01	28.49
0.7	.85	.70	.89	1.02	28.59
0.8	.85	.70	.89	1.02	28.59

0.9	1.14	0.96	0.97	1.01	27.80
0.95	1.14	0.96	0.97	1.00	27.80
1.00	1.14	0.94	0.98	0.98	27.84

Table S4. Occupancies for each Cl-atom, isotropic refinement, no restraints, 63 parameters.

Start occupancy	End occupancy				R1
	Cl1	Cl2	Cl3	Cl4	
0.1	.56	.67	.74	.77	32.99
0.5	0.56	0.60	0.74	0.69	33.01
0.6	1.04	1.02	1.01	1.06	30.11
0.7	1.04	1.02	1.01	1.06	30.11
0.8	1.02	0.97	1.01	1.04	30.13
0.9	1.02	0.97	1.01	1.04	30.13
0.95	1.02	0.97	1.01	1.04	30.13
1.00	1.04	1.00	1.10	1.08	30.15

Dynamical refinement against 3D ED data

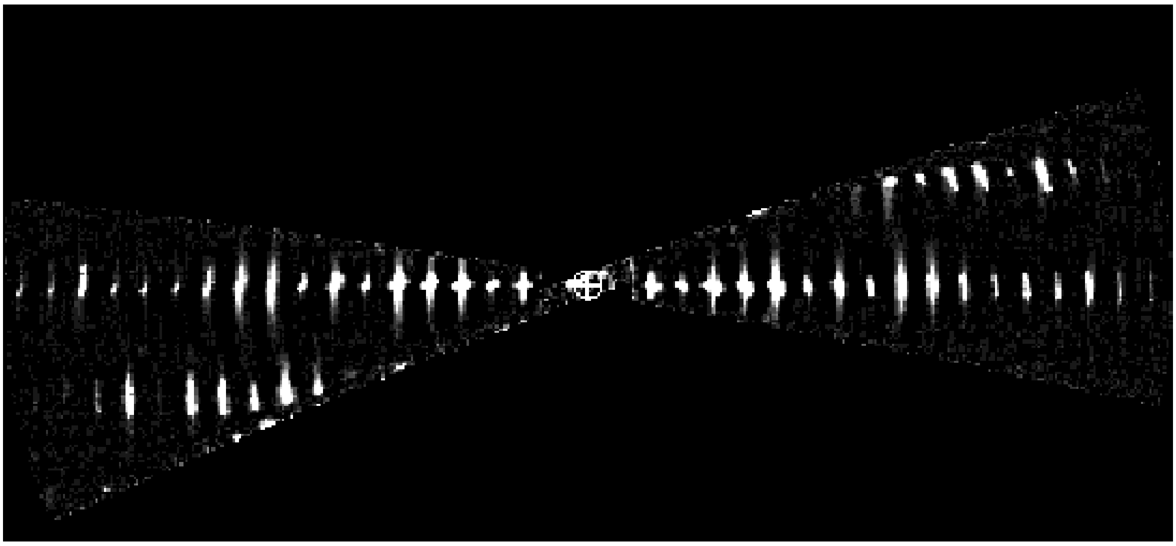

Figure S3 hk0 plane of the reciprocal volume reconstructed from 3D ED data using PETS2 (Prague, Czech Republic).

FIDEL fits

Table S5. FIDEL fits. General settings and parameters for all fits: static background correction, Lorentz profile function with static constant FWHM = 0.425° (only optimized for final structure candidates). S_{12} similarity values were calculated comparing the background-corrected experimental data to the simulated pattern over the 2θ range 3 - 70°, using values of the neighbouring range parameter l as given in the table or in brackets. Reference similarities S_{12}^0 denote the limit of $S_{12}(l)$ as l approaches 0 over the full range of the experimental pattern, comparing the patterns including the background (unless the use of the background-corrected data is explicitly indicated by the superscript "bc").

	**Local fit**	**Local fit**	**Regional fit**	**Regional fit**
**SG, Z**	*C* 2/*m*, *Z* = 2	*P* 2_1, *Z* = 2	*C* 2/*m*, *Z* = 2	*P* 2_1, *Z* = 2

**Start structure or configured range**				
a / Å	17.685	17.685	15.685 - 19.685	15.685 - 19.685
b / Å	25.918	25.918	23.918 - 27.918	23.918 - 27.918
c / Å	3.833	3.833	1.833 - 5.833	1.833 - 5.833
β / °	95.05	95.05	75.05 - 115.05	75.05 - 115.05
m_x (in fractional coords)	0	0.25	0	0.05 - 0.45
m_y, m_z (in fractional coords)	0	0	0	-0.20 - 0.20
ϕ_y / °	0	0	-20 - 20	-20 – 20
ϕ_x, ϕ_z / °	0	0	0	-20 – 20
V / Å^3	1750.1	1750.1	1564.7 - 2347.1	1564.7 - 2347.1
**FIDEL fits**				
Number of fitted parameters	5	10	5	10
Initial neighb. range *l* / °	0.5	0.5	1.0	1.0
Smallest neighb. range *l* / °	0.1	0.1	0.6	0.6
Trial starting structures	1	1	24962	119990
S_{12} (start)	0.77365	0.77365	0.12557 - 0.92689	0.09036 - 0.9198
**Fitted structures:**				
Number of local optimisations	1	1	357	625
Number of post-optimisations	1	1	150	150
V / Å^3 (all opt.)			1542.16 - 1921.68	1529.99 - 1893.57
$S_{12}^{0,bc}$ (post-opt.)	0.88750	0.89708	0.79448 - 0.90246	0.77418 - 0.91026
Candidates (clustered)	1	1	39	76
**Best / final result:**				
a / Å	17.6232	17.7350	17.8241	17.7878
b / Å	26.0814	26.0806	26.0534	26.0490
c / Å	3.7830	3.7830	3.9169	3.9157
β / °	95.8351	95.4830	99.531	99.221
V / Å^3	1729.80	1741.78	1793.82	1790.91
$S_{12}(l = 0.1)$	0.91356	0.92906	0.92561	0.93480
S_{12}^0	0.99302	0.99382	0.99381	0.99383
R_{wp} / %	14.42	14.68	13.62	15.22
FWHM (opt.) / °	0.441	0.329	0.403	0.360
**Alternative result:**				
a / Å			18.054	17.863
b / Å			26.060	26.023
c / Å			3.747	3.819
β / °			95.380	96.885
V / Å^3			1755.17	1762.47
$S_{12}(l = 0.1)$			0.90419	0.92671
S_{12}^0			0.99259	0.99359
R_{wp} / %			14.62	15.28
FWHM (opt.) / °			0.425	0.359

DFT calculations

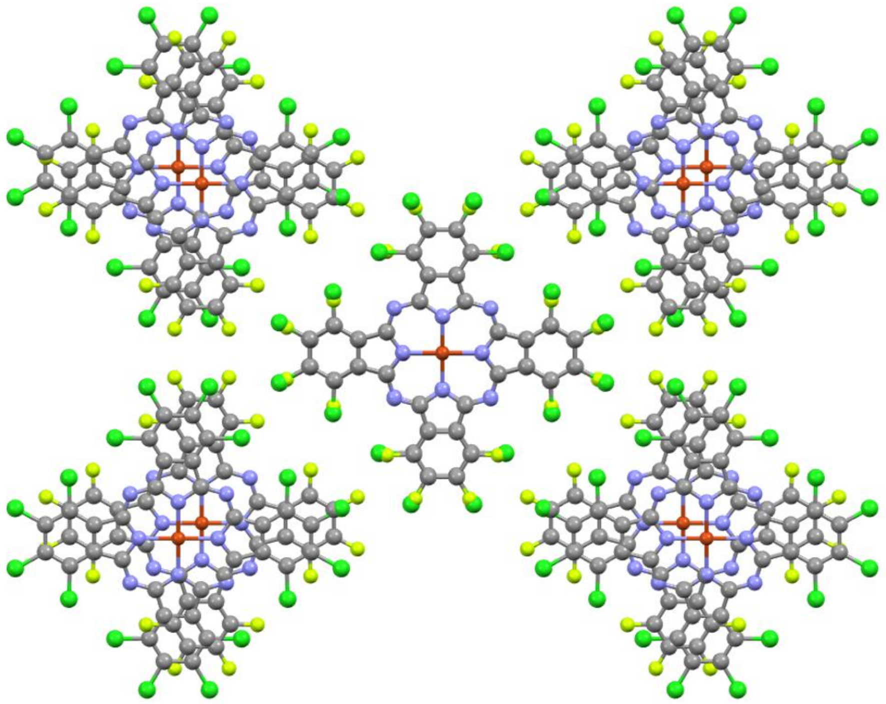

Figure S4 Overlay of the parallel packed $CuPcCl_{16}$ structure with the hypothetical $CuPcF_{16}$ (*) structure after energy minimization. Chlorine atoms are green, fluorine – yellow.
